# Implementation of a new prenatal care model to reduce office visits and increase connectivity and continuity of care: protocol for a mixed-methods study

**DOI:** 10.1186/s12884-015-0762-2

**Published:** 2015-12-02

**Authors:** Jennifer L. Ridgeway, Annie LeBlanc, Megan Branda, Roger W. Harms, Megan A. Morris, Kate Nesbitt, Bobbie S. Gostout, Lenae M. Barkey, Susan M. Sobolewski, Ellen Brodrick, Jonathan Inselman, Anne Baron, Angela Sivly, Misty Baker, Dawn Finnie, Rajeev Chaudhry, Abimbola O. Famuyide

**Affiliations:** Robert D. and Patricia E. Kern Center for the Science of Health Care Delivery, Mayo Clinic, 200 1st Street SW, Rochester, MN 55905 USA; Department of Health Sciences Research, Mayo Clinic, 200 1st Street SW, Rochester, MN 55905 USA; Obstetrics Division, Mayo Clinic, 200 1st Street SW, Rochester, MN 55905 USA; Office of Risk Management, Mayo Clinic, 200 1st Street SW, Rochester, MN 55905 USA; Practice Administration, Mayo Clinic, 200 1st Street SW, Rochester, MN 55905 USA; Primary Care Internal Medicine, Mayo Clinic, 200 1st Street SW, Rochester, MN 55905 USA; Center for Innovation, Mayo Clinic, 200 1st Street SW, Rochester, MN 55905 USA

**Keywords:** Prenatal care, Patient-focused care, Health services research, Program evaluation

## Abstract

**Background:**

Most low-risk pregnant women receive the standard model of prenatal care with frequent office visits. Research suggests that a reduced schedule of visits among low-risk women could be implemented without increasing adverse maternal or fetal outcomes, but patient satisfaction with these models varies. We aim to determine the effectiveness and feasibility of a new prenatal care model (OB Nest) that enhances a reduced visit model by adding virtual connections that improve continuity of care and patient-directed access to care.

**Methods and design:**

This mixed-methods study uses a hybrid effectiveness-implementation design in a single center randomized controlled trial (RCT). Embedding process evaluation in an experimental design like an RCT allows researchers to answer both “Did it work?” and “How or why did it work (or not work)?” when studying complex interventions, as well as providing knowledge for translation into practice after the study. The RE-AIM framework was used to ensure attention to evaluating program components in terms of sustainable adoption and implementation.

Low-risk patients recruited from the Obstetrics Division at Mayo Clinic (Rochester, MN) will be randomized to OB Nest or usual care. OB Nest patients will be assigned to a dedicated nursing team, scheduled for 8 pre-planned office visits with a physician or midwife and 6 telephone or online nurse visits (compared to 12 pre-planned physician or midwife office visits in the usual care group), and provided fetal heart rate and blood pressure home monitoring equipment and information on joining an online care community.

Quantitative methods will include patient surveys and medical record abstraction. The primary quantitative outcome is patient-reported satisfaction. Other outcomes include fidelity to items on the American Congress of Obstetricians and Gynecologists standards of care list, health care utilization (e.g. numbers of antenatal office visits), and maternal and fetal outcomes (e.g. gestational age at delivery), as well as validated patient-reported measures of pregnancy-related stress and perceived quality of care. Quantitative analysis will be performed according to the intention to treat principle. Qualitative methods will include interviews and focus groups with providers, staff, and patients, and will explore satisfaction, intervention adoption, and implementation feasibility. We will use methods of qualitative thematic analysis at three stages. Mixed methods analysis will involve the use of qualitative data to lend insight to quantitative findings.

**Discussion:**

This study will make important contributions to the literature on reduced visit models by evaluating a novel prenatal care model with components to increase patient connectedness (even with fewer pre-scheduled office visits), as demonstrated on a range of patient-important outcomes. The use of a hybrid effectiveness-implementation approach, as well as attention to patient and provider perspectives on program components and implementation, may uncover important information that can inform long-term feasibility and potentially speed future translation.

**Trial registration:**

Trial registration identifier: NCT02082275

Submitted: March 6, 2014

**Electronic supplementary material:**

The online version of this article (doi:10.1186/s12884-015-0762-2) contains supplementary material, which is available to authorized users.

## Background

Prenatal care is a key preventive health service used in developed countries around the world. By providing expectant mothers with regular health evaluations and information about the course of the pregnancy, labor, birth, and parenthood, prenatal care aims to reduce the risk of unfavorable pregnancy and birth outcomes. Most prenatal care occurs in the setting of routine office visits. In the U.S., the American College of Obstetricians and Gynecologists (ACOG) recommends a uniform prenatal visit schedule comprised of approximately 14 visits: every four weeks up to 28–32 weeks of gestation, then every two weeks up to 36 weeks, and finally weekly until birth [1]. This rhythm of care has been codified largely based on tradition that is informed by a plan of care directed at the detection of risks such as hypertensive disorders of pregnancy. However, research does not necessarily support high numbers of visits for the majority of low-risk pregnancies [2, 3]. Indeed, there is a wide variation in visit schedules across countries, and higher numbers of visits do not necessarily correspond with better outcomes [3]. While there is no doubt that prenatal care is important to maternal and fetal health, there is limited evidence of an exposure-response relationship between visit frequency and outcomes [4].

In 1989, the Expert Panel on the Content of Prenatal Care issued recommendations for prenatal care that were based on expert review and consensus of the limited best available evidence [5]. These included a recommendation that the visit schedule be flexible and based on the needs of each expectant mother, for example more visits for nulliparous and high-risk women and fewer visits for multiparous and low-risk women. Subsequently, several studies have considered the effectiveness of reduced antenatal care schedules. Most have found that a reduced schedule of visits among low-risk women could be implemented without increasing adverse maternal or fetal outcomes such as preterm delivery, preeclampsia, and low birth weight [6–12], although there is mixed evidence on perinatal mortality [11, 13]. Some studies found patient satisfaction was unchanged [6] or improved [9, 10], but a larger number suggest that patient satisfaction decreases with lower numbers of visits [7, 8, 11, 14].

Reasons for reduced satisfaction are unclear. Sikorski et al. found patients receiving a reduced schedule were less likely to feel listened to and more likely to want more time to talk at visits [7]. More than 50 % of the study participants felt that some gaps between visits were too long. Novick’s qualitative assessment of women’s preferences for prenatal care included continuity of care, flexibility, comprehensiveness of care (including access to group discussions with other pregnant women), developing meaningful relationships with professionals, and becoming more active participants in care [15]. Structural barriers like inconveniences of office visits can limit women’s access to prenatal care [16].

Since the expert panel’s report 25 years ago, and despite general consensus on the safety and effectiveness of a reduced visit schedule on maternal and fetal outcomes, new models that address women’s preferences have not been widely adopted [17]. Some investigators suggest that the effectiveness of prenatal care should not be defined solely in terms of risk assessments and number of prenatal office visits, but rather in terms of the content of the care [18, 19]. New models of care that address access and continuity of care while reducing the burden of pre-planned office visits could result in increased patient satisfaction. However, there is limited research on new models that go beyond adaptation of the visit schedule.

### Developing a new model of care

In 2011, members of the Obstetrics Division at Mayo Clinic (Rochester, MN), together with the Mayo Clinic Center for Innovation (CFI) design team, developed a new model of care for low-risk pregnancies that reduced office visits while increasing virtual connections with nurses and providers. This prenatal care program (OB Nest) was to be based on proactive and direct support from a nursing team that could meet the on-demand needs of expectant mothers as they arise, redesigning the need for and timing of planned on-site appointments with providers while increasing expectant mothers’ experience of and satisfaction with their care.

Their theory of action was that the traditional model of prenatal care predates methodological and technological advances in pregnancy monitoring and communications, assuming a rhythm of care dependent on face-to-face visits that preceded an appreciation for the busy lives of women. Furthermore, it engendered the idea that the prenatal office visit is the sole intended point of contact for expectant mothers with their care team, shifting the locus of control to the provider as in disease treatment models, thus medicalizing the pregnancy experience. Pregnant women may associate the high frequency of visits in the traditional model with safe pregnancy outcomes. In developing the OB Nest care model, the team hypothesized that empowering low-risk pregnant women to retain more control of their prenatal care shifts their health care delivery from a sickness model to a wellness model.

**Fig. 1:**
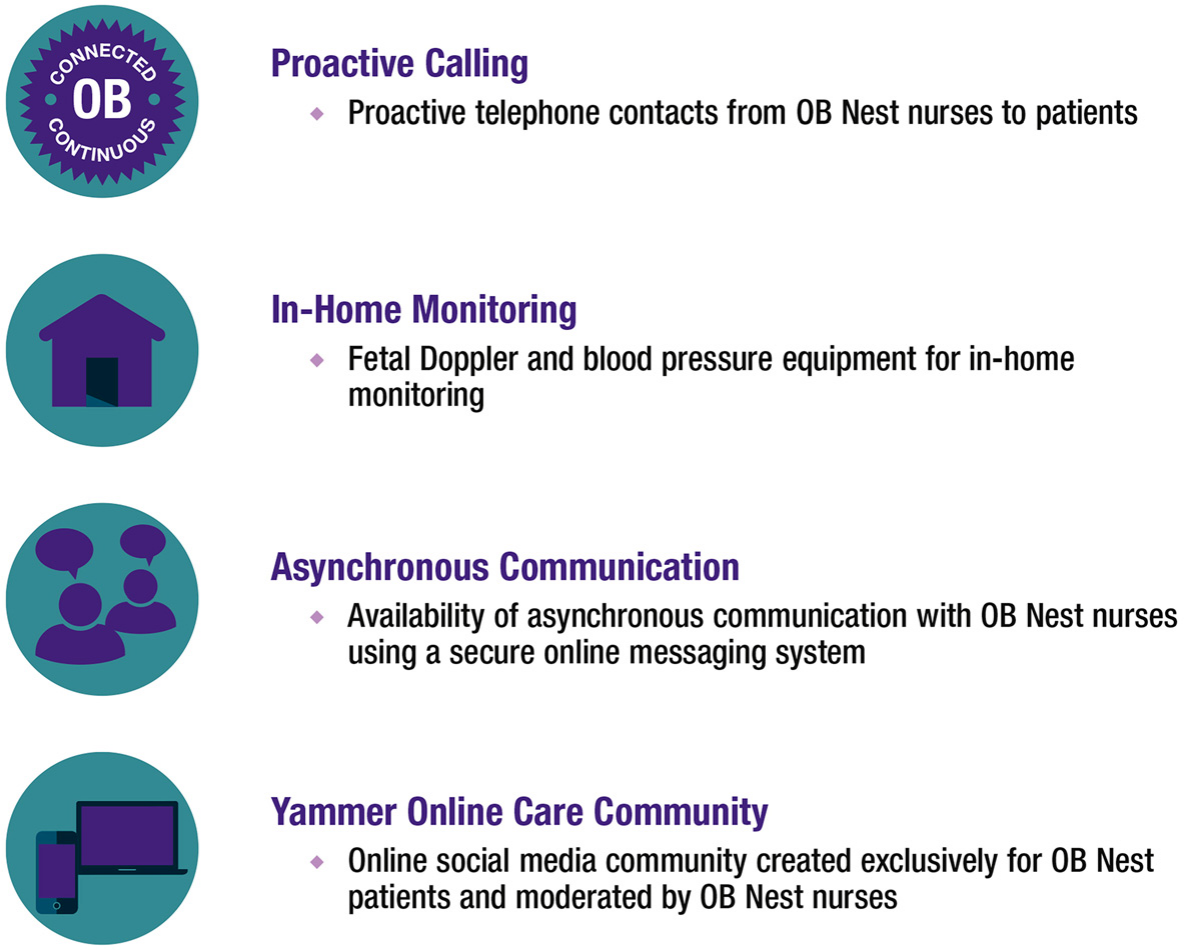
OB Nest intervention components

As a result, the team designed and rapid-tested potential components of the new program (summary information available at www.mayo.edu/center-for-innovation/projects/ob-nest/). The following were selected for inclusion in OB Nest: Online Care Communities, At-Home Measurement, Video Appointments (with the option of phone where video is not available), and Proactive Calls. This paper reports the study protocol for the evaluation of the OB Nest program (Fig. 1).

### Study purpose

The purpose of this study is to determine the effectiveness of the OB Nest program, compared to usual prenatal care in this practice, and to evaluate the implementation process. The primary research and evaluation questions are as follows:

1. Does OB Nest improve patient-reported satisfaction with care, as well as pregnancy-related stress and perceived quality of prenatal care?
2. Does OB Nest reduce the rate of in-clinic health care utilization without impacting maternal/fetal outcomes or fidelity to the process of care standards specified by ACOG?
3. Can OB Nest be implemented as intended during the study, and can it be feasibly adopted into practice?

## Methods and design

### Study design

This study uses a hybrid effectiveness-implementation design in a single center randomized controlled trial (RCT) to compare the OB Nest intervention to usual care among low-risk pregnant women. Specifically, this is a Type 1 hybrid design as described by Curran and colleagues whereby the primary aim is a test of an intervention’s clinical effectiveness but there is also an a priori aim related to observing and gathering data on implementation [20]. Embedding process evaluation in an experimental design like an RCT allows researchers to answer both “Did it work?” and “How or why did it work (or not work)?” when studying complex interventions like those in health services [21]. This approach also has the potential to speed clinical uptake by delivering information on the intervention’s effectiveness in the study setting alongside data to inform external validity, such as the barriers and facilitators to widespread implementation [20, 22].

Mixed methods are well-suited for these types of implementation studies, where quantitative methods are used to test effectiveness and qualitative methods are used to understand process, participant perspectives (practitioners and consumers), and the intervention context [23, 24]. In this study, the quantitative methods are dominant *(QUAN),* supporting the primary aims of the study, and the qualitative methods are less dominant *(qual). QUAN* and *qual* data collection, as well as data analysis, will occur concurrently or simultaneously in a parallel mixed design [24].

### Guiding framework

We used the RE-AIM framework to guide the evaluation plan, including the selection of mixed methods [25]. This framework has been developed specifically to address questions of implementation, effectiveness, and external validity in studies conducted in real-world settings. The dimensions of the framework include: Reach (how willing the targeted population is to participate in the intervention), Effectiveness (the impact of the intervention on outcomes), Adoption (whether the intervention can be adopted with ease and minimal modifications), Implementation (what are the special issues and barriers to implementation), and Maintenance (can the intervention be maintained and will the impact continue).

To assess Reach, we will compare patient characteristics (eg, age) between those who agreed to participate in OB Nest and those who declined participation. This will allow us to measure participation and representativeness of the trial. We will also record the reasons that decliners gave to the study coordinator, and for ineligible patients we will track reasons for ineligibility. We will use a number of patient-reported outcome measures and clinical or practice-level outcome measures to assess Effectiveness on study outcomes including satisfaction, quality of care, stress, safety and utilization. To assess Adoption, we aim to understand the extent to which patients use OB Nest components, as well as the organizational or individual barriers and facilitators to provider and staff participation in OB Nest, including values and preferences. We anticipate that patients will vary in adoption of the various OB Nest components as the care model is meant to be patient-driven. To assess Implementation, we will seek to determine whether the intervention has been implemented as planned, and potential issues that need immediate adaptation before full implementation post-study would be possible. Considering the length of the study, we will not be able to fully assess Maintenance, but data on effectiveness and implementation should provide preliminary insights as to whether we can maintain this intervention post-study. Table 1 outlines the outcomes and quantitative and qualitative data collection methods, as described below, using the RE-AIM framework. This approach is well suited for the evaluation of complex, multi-component interventions like OB Nest where the aim is to evaluate not only overall program effectiveness but the potential for successful translation and sustainability.

**Table 1:** Data collection method and outcome by RE-AIM criteria

RE-AIM criteria	Outcome	Method	Type of data
Reach	Participant and decliner characteristics, decliner reasons, and ineligibility reasons	Administrative data	QUAN
Effectiveness	Utilization (eg, in-office visits)	Administrative data	QUAN
	Maternal/fetal outcomes (eg, gestational age at delivery)	Administrative data	QUAN
	Receipt of standard prenatal testing and care (ACOG)	Administrative data	QUAN
	Patient stress	Questionnaires	QUAN
	Patient satisfaction and perceived quality of care	Questionnaires	QUAN
		Patient interviews and focus groups	qual
Adoption	Extent to which participants use intervention components	Patient interviews and focus groups	qual
		Provider and staff interviews and focus groups	qual
		Administrative data	QUAN
	Organizational or individual barriers to intervention use	Provider and staff interviews and focus groups	qual
		Patient interviews and focus groups	qual
		Document review	qual
Implementation	Implementation as planned, and issues in implementation	Patient interviews and focus groups	qual
		Provider and staff interviews and focus groups	qual
		Document review	qual
Maintenance^a^			

Abbr: QUAN = quantitative, qual = qualitative, ACOG = American College of Obstetricians and Gynecologists

^a^Effectiveness data and implementation data will inform maintenance, but maintenance will not be fully assessed due to the study length

### Setting

This study will recruit individuals from the Obstetrics Division at Mayo Clinic (Rochester, MN), a tertiary Midwest teaching hospital with a delivery rate of 2400 women annually. The practice consists of 14 full-time obstetricians, 5 maternal fetal medicine specialists, 10 certified nurse midwives (CNM), and 10 family medicine physicians. A 20 bedded level 3 neonatal intensive care unit serves as a regional referral center and is staffed by 4 full-time neonatologists. The usual model of prenatal care includes a set schedule of 12 pre-planned in-office visits with a physician or a certified nurse midwife (sometimes referred to as “providers” in this article). CNMs in this practice are registered nurses with Master of Science degrees in midwifery, and they provide a full range of prenatal care and labor and delivery services. Registered nurses in the department assist with patient care and provide patient education during provider office visits and in patient education courses. They also provide support to patients by telephone.

### Sample size

On average 2400 pregnant women receive prenatal care from the Obstetrics Division in a 12-month period, and about 60 % of these are considered to be low-risk pregnancies. We will enroll 300 patients in this study (150 patients per arm). This accounts for a withdraw/post-randomization exclusion rate as high as 10 %, so the effective sample size can be as low as 135 patients/arm. Sample size calculations are displayed in Table 2.

**Table 2:** Sample size calculations

Outcome	Estimated rate for standard of care^a^	Improvement to be tested/expected	Power
Patient-reported outcomes			
Satisfaction with care	108.4 (SD 14.4)	115.6	98 %
Perceptions of quality of care	83.9 (SD 22.8)	91.9	86 %
Prenatal stress	12.9 (SD 7.1)	16.5	98 %
Maternal/fetal outcomes and standards of care			
Low birth weight	11 %	6 %	24 %
Gestational age at delivery	38.9 (SD 2.5)	40.2	99 %
Standard of prenatal care (measured as receiving at least 20 out of 24 items from the ACOG standards of prenatal care list)	90 %	95 %	30 %
Health care utilization			
Number of antenatal visits	14.7 (SD 4.2)	12	99 %

Abbr: ACOG = American Congress of Obstetricians and Gynecologists

^a^All calculations 2-sided with alpha of 0.05. Continuous outcomes compared to a half of standard deviation increase

We will interview up to 40 patients and conduct approximately 4 patient focus groups of 10–12 participants each. We will also interview physicians (*n* = 6) and conduct focus groups with midwives (*n* = 9), nurses (*n* = 7), and desk staff/clinical assistants (*n* = 10) at three points in time. This sample size is within standards for qualitative research, but we will increase the sample size if needed to achieve saturation in identifying themes.

### Participant recruitment and randomization

Patients will be recruited in clinic at the typical 90-min appointment scheduled with a registered nurse at approximately 8 weeks of pregnancy. The targeted patient population for this study will be low-risk patients. The study coordinator will ascertain the patient’s interest in participating in the study and study eligibility, and a written consent form will be shared with eligible patients. Inclusion criteria include age 18 to 36 years at time of enrollment (older patients excluded under the definition of advanced maternal age as a criterion for risk), documented gestational age less than 13 weeks, pregnancy documented as low risk without a concurrent medical or obstetric complication, ability to read and understand English, and ability to provide informed consent. Women will be excluded if they have any of the conditions listed in the patient enrollment exclusion criteria [see Additional file 1]. Randomization will be conducted using a dynamic allocation [26] algorithm minimizing imbalances across multiple assigned stratification factors. Patients will be stratified by enrolling age (≥35 vs. <35), BMI (>30 vs. ≤30) and parity (0 vs. 1+). A physician will confirm OB Nest patients’ risk assessment at the new OB return appointment at approximately 12 weeks gestation, consistent with current practice workflow. Patients determined to be high-risk at that time will be removed from the study (post-randomization exclusion). We expect exclusions to be minimal due to the clarity of inclusion/exclusion criteria and the availability of physicians as needed for patient eligibility questions during the initial 8-week visit.

Patients randomized to the OB Nest program will be assigned to a dedicated nursing care team comprised of three registered nurses, and they will be scheduled for 8 pre-planned office visits with a provider (physician or CNM) compared to 12 in the usual care group, as shown in Table 3. The 4 office visits will be replaced with connected care visits (phone or online patient portal) with one of the nurses, whom patients can also contact with on-demand questions. There will also be two additional connected care visits in the OB Nest schedule, making total contacts greater in the OB Nest group, even though office visits are reduced. An electronic population management registry was developed on the Caradigm platform for care team management of OB Nest patients. Patients will be listed for the week that the nurse team is to contact them, and lab and imaging tests that have been completed will populate the registry in real time from the electronic medical record. The registry will also send data (task lists) to the clinical team daily based on the protocol of care designed for this initiative.

**Table 3:** Comparison of visit schedule by week of gestation, usual care and OB Nest groups

Week	Usual care group	OB nest group	Care provided (both groups)
1-7	Drop-in pregnancy education	Drop-in pregnancy education	Interactive self-education
8	New OB office visit with registered nurse/CNM	New OB office visit with registered nurse/CNM	Individualized pregnancy education, bedside ultrasound, history, lab ordering, discuss genetic options
12	New OB office visit with physician/CNM	New OB office visit with physician	Physical exam including pap if needed and review plan of care
16		Connected care visit with registered nurse^a^	Education and review of care
18	Ultrasound, registered nurse visit	Ultrasound, registered nurse visit	Ultrasound; mid-pregnancy education
24	Routine OB office visit with physician/CNM	Connected care visit with registered nurse^a^	Routine^b^
28	Routine OB office visit with physician/CNM	Routine OB office visit with physician/CNM	Routine^b^ (lab ordering)
33	Routine OB office visit with physician/CNM	Connected care visit with registered nurse^a^	Routine^b^
36	Routine OB office visit with physician/CNM	Routine OB office visit with physician/CNM	Routine^b^ (lab ordering)
38	Routine OB office visit with physician/CNM	Connected care visit with registered nurse^a^	Routine^b^
39	Routine OB office visit with physician/CNM	Routine OB office visit with physician/CNM	Routine^b^
40	Routine OB office visit with physician/CNM	Connected care visit with registered nurse^a^	Routine^b^
41	Routine OB office visit with physician/CNM; registered nurse visit; ultrasound	Routine OB office visit with physician/CNM; nurse visit; ultrasound	Over 40 weeks bi-weekly monitoring
1 week Postpartum		Connected care visit with registered nurse^a^	Education and review of care
8 weeks Postpartum	Routine OB visit with physician/CNM	Routine OB Office visit with physician/CNM	
Total visits to clinic	12	8	

Abbr: CNM = Certified Nurse Midwife

^a^Connected care visits with nurses are scheduled contacts by phone or online patient portal

^b^Routine = Check maternal blood pressure, maternal weight, and fetal heart rate, and provide education

Compared to usual care, OB Nest patients will have 4 fewer office visits. However, total scheduled contacts during pregnancy are greater in the OB Nest group. In addition to changing provider office visits with nurse virtual connected care visits, there are additional nurse contacts at 16 weeks and one week postpartum

Patients will be able to request additional in-office visits if desired and providers may suggest additional in-office visits to monitor maternal or fetal concerns. Laboratory tests, imaging, and standardized evaluations will be conducted in both arms of the study. OB Nest patients will be invited to participate in an online, nurse-moderated community with other OB Nest participants, where they can seek advice and support from their peers. Finally, OB Nest patients will be provided a fetal Doppler and automated blood pressure machine for home use, along with training on how to use the equipment. This equipment will allow patients to monitor their health and provide an opportunity for others to be involved in these experiences outside of office visits. Patients will be asked to keep a weekly journal of weight, fetal heart rate, and blood pressure readings and report those to nurses at the connected care visits.

### Data collection

#### Quantitative (QUAN)

Patient characteristics will be collected for patients in both arms (*n* = 300) at time of enrollment through self-report and medical record review including: age, race/ethnicity, education level, marital status, body mass index (BMI), due date, parity, previous miscarriage, previous C-section, and insurance. The following patient-reported outcome instruments, administered by email or by mail if requested, will be used to provide data on locus of control (collected as a measure of participant characteristics at baseline), satisfaction with care, quality of care, and stress related to pregnancy in both arms:

- The validated Fetal Health Locus of Control Scale will be used at the time of enrollment to assess patients’ feelings of control in their pregnancies [27]. The scale contains 18 items, with 6 questions per subscale that address internal control, control by health professionals and control by God/fate/chance. Points will be summed and reported on each subscale.
- Patient satisfaction (primary outcome) with care will be assessed using the validated 16-item Satisfaction subscale, which is scored on a 5-point scale from ‘very dissatisfied’ to ‘very satisfied.’ The scale will be collected at the 36-week assessment [28]. Responses will be summed and converted to a 0–100 point scale where higher scores are indicative of a higher degree of satisfaction.
- Patient perceptions of quality of care will be assessed using the validated Prenatal Interpersonal Processes of Care (PIPC) scale [29]. The 30 items cover 3 subscales that address communication, decision making and interpersonal style. The scale will be collected at the 36 week assessment. Responses will be converted to a 0–100 scale for each subscale.
- Stress will be evaluated using the validated PreNatal Maternal Stress (PNMS) scale [30]. The 9-item scale will be emailed to patients at approximately 14 weeks gestation and again with additional items at approximately 24 weeks and 26 weeks gestation. Scores will be summed and averaged where higher scores are indicative of a higher degree of stress.

Data from the medical record will be abstracted to capture utilization, fidelity to standards of prenatal care, and maternal/fetal outcomes. The time frame for collection will be from time of enrollment to the 6–8 week postpartum appointment. Medical record review will be conducted to capture all in-office obstetrics visits, assessments in the out-patient OB triage center, and hospitalizations. It will also be used to track clinically-important outcomes including incidence and dates of pre-eclampsia/eclampsia, hypertension, anemia, urinary tract infection, hematocrit, infection, and loss of pregnancy. Assessment at the time of delivery will include maternal weight gain, type of delivery, length of stay during hospitalization, gestational age at delivery, birth weight, and Apgar scores. To assess compliance with standard processes of care, study staff will review the medical record for patients in both arms against a list of ACOG standards of prenatal care, for example depression screening, HIV testing, prenatal vitamin/iron supplement prescription, offering influenza vaccine, and screening for gestational diabetes.

Utilization data from the medical record will be supplemented by data from an internal data tracking system used to detail OB Nest patient and nursing contact, including numbers of phone calls, on-line visits, and conversations through the secure online patient portal. Nurses will also track time spent on OB Nest patient care in a department time tracking system. These data, along with average assessments of time spent on in-office visits (provider, rooming nurse and desk staff) and FTE calculations provided by department financial staff, will be used to assess staffing demands in both arms.

#### Qualitative (qual)

Qualitative one-on-one interviews and asynchronous online focus groups (online discussion boards where individuals are not required to participate in real-time) will be used to gather data on perceptions of quality and satisfaction. OB Nest participants will be sampled from a list of those who indicated interest during the consent process. We hypothesize that a woman’s experience will differ by whether she is a first time mother or not, so we will stratify based on parity.

We will complete individual semi-structured interviews with up to 40 OB Nest patients. Interviews, conducted by a trained qualitative interviewer, will be approximately 30-min in length and typically conducted by phone. Because we want to interview patients after significant exposure to intervention activities, we will aim to conduct the interview when the patient reaches 28 and 32 weeks gestation. Patient focus group data collection (4 groups of 10–12 patients each) will begin after interview data collection. The asynchronous online focus groups will be open for a week, with new topics and questions posted each day by the moderator (a member of the research team). The topics and questions will be semi-structured and based on the initial analysis of the individual patient interviews. The online asynchronous nature of the groups means participants will be able to sign on at a time and place that is convenient for them.

Interviews and discussions with providers, nurses, and desk staff/clinical assistants will be used to gather information on staff perceptions of the intervention and its implementation. We will invite all physicians, nurse midwives, nurses, desk staff, and clinical assistants involved in the study to provide qualitative data. Data collection will happen at three time points during the study: baseline (first month of the study), mid-point, and end of study. Interview guides will be semi-structured and will include questions based on the RE-AIM framework, as well as Normalization Process Theory, which is a sociological theory of the implementation, embedding, and integration of new technologies and organizational innovations in complex settings [31, 32]. This theory informed questions on how providers and staff understand the purpose of OB Nest and its value in their practice, how they perceive the impact it will have on their work, whether appraisal has led to changes in practice, and whether people are engaged in driving the initiative forward, for example.

Finally, document review, which will involve capturing the written documents from OB Nest implementation (eg, presentations and memos about OB Nest), will further help us understand how it was perceived and what contextual factors may have shaped implementation.

### Analysis plan

#### Quantitative (QUAN)

Quantitative analysis will be performed according to the intention to treat principle, including all participants in the arm to which they were randomized, regardless of whether they received the intervention assigned or crossed over to the other treatment arm. Baseline characteristics will be reported in the study results with continuous values being reported as means and standard deviations and categorical values reported as counts and frequencies and compared between study arms using t-tests and chi-squared tests, respectively.

We will use standard techniques appropriate for participant level randomized trials, with each outcome compared between study arms using t-tests for continuous outcomes and chi-square tests for dichotomous outcomes. Any baseline imbalances (*p* < 0.05) will be explored as a possible factor to adjust for when the outcome measures are analyzed. We will use predetermined criteria for missing data when calculating scores on patient-reported surveys. The comparison of outcomes will be considered significantly different between arms at a *P* < 0.05.

Participants who move into a high risk category after 12 weeks will stay on study and be evaluated in the arm to which they were randomized. If more than 10 % of patients choose to crossover from intervention to control, a sensitivity analysis will be conducted. Trial enrollment, completeness of data collection, and fidelity of follow-up procedures will be reviewed and reported during study team meetings which will be conducted on a monthly basis. Analysis of quantitative data will be performed at the close of the study.

#### Qualitative (qual)

Audio-recorded interviews will be transcribed verbatim, de-identified, and verified against actual recordings by study staff. In-person focus groups will be audio-recorded and both the moderator and an observer will complete observational notes for analysis. The analysis team will review these audio recordings for completeness of captured data in the notes. Online focus group transcripts will be captured electronically.

We will use methods of qualitative thematic analysis with interview and focus group data, as well as in the document analysis [33, 34]. This approach begins with familiarization with the data. The next step is to identify key issues and concepts that will form the basis of a coding framework. This framework will be independently applied to the interview and online focus group transcripts by two members of the research team who will then meet to come to consensus on coding. Qualitative analysis software (NVivo 10.1, QSR International Pty Ltd.) will be used to facilitate data organization. Coded data will be reviewed by the research team who will begin to identify thematic findings in the data, comparing the data from these perspectives of participants against observational notes and documents to gain a rich picture of context and experience. Data will be analyzed within and across groups (patients, physicians, midwives, nurses, and desk staff). This process will happen in three stages (baseline, mid-point, and end of study), with each stage building on knowledge from earlier stages. Throughout this process we will engage the clinical study team for input on findings and interpretation.

#### Mixed methods

One function of mixed methods is expansion, or using one method to explain the results of another [23]. At the end of the study, mixed methods analysis will involve comparing results to increase understanding, specifically the use of qualitative data to lend insight to quantitative findings. This includes patient satisfaction and quality of care, as patient perspectives from interviews and focus groups will be analyzed against validated questionnaires to see if these data can help inform findings. Qualitative data on implementation, specifically data on how patients and providers/staff use the intervention and how it operated in practice, may also complement findings on effectiveness. This type of mixed methods analysis will serve as methods triangulation (checking the consistency of findings generated by different methods) [24, 33].

### Ethical considerations

All participants will complete written informed consent and be told that they can withdraw from the study at any time. This research was approved by the Mayo Clinic Institutional Review Board (IRB #13-009513) and is registered in ClinicalTrials.gov (trial registration identifier: NCT02082275).

### Reporting of study findings

We will adhere to the CONSORT guidelines [35, 36] for reporting RCTs to transparently report study results and ensure that sufficient information is included to allow for assessment of the study’s internal and external validity [35, 36]. We will use the consolidated criteria for reporting qualitative research (COREQ) in reporting qualitative findings, including the search for negative cases when determining saturation or the point at which no new themes are emerging from the data [37].

## Discussion

In spite of a substantial body of evidence that demonstrates the safety of reduced visit prenatal care models, practices have been slow to adopt this model of care [17]. This reluctance may in part be due to the results of studies that show lower patient satisfaction with reduced visit schedules [7, 8, 11, 14]. A new model of care that reduces pre-planned office visits but also increases virtual or other connections with staff and other pregnant women, leveraging new technologies for communications and connectedness, could address lingering concerns about patient satisfaction on issues of continuity of care and feelings of being supported. This wellness-focused model could also empower women in directing their care. Early pilot testing of OB Nest supported the theory of action around how the program should work to affect outcomes, for example that empowering women in their care would increase satisfaction, but the team needed evidence of OB Nest’s effectiveness before moving forward with practice change.

Early discussions between research team members started around designing a RCT to test not only effectiveness on fetal and maternal outcomes currently in the literature, but also on patient-important measures like satisfaction. This rigorous approach would make important contributions to the literature on reduced visit models by including an expanded set of outcomes that would address lingering questions. However, the team quickly moved toward a hybrid effectiveness-implementation approach with a priori aims that would uncover important information about how the intervention was actually implemented in a real world setting. The RE-AIM framework focused our efforts on understanding a range of outcomes including the feasibility of implementation, which is a critical step in determining long-term feasibility and external validity, ultimately facilitating translatability of evidence into practice.

### Limitations and strengths

Strengths of the study include the use of a range of outcomes currently not well-represented in the literature, the hybrid study design that embeds a process evaluation in an experimental design, and the use of mixed methods. Previous studies have used one approach or the other—an experimental design to test clinical outcomes or qualitative methods to understand participant perspectives—but this study has the benefit of combining both into a more complete picture of both “Did it work?” and “How or why did it work (or not work)?” Without this more complete analysis, implementation of new models of care may continue to falter.

The study protocol does come with some risks to successful execution. One of these may occur when researchers or stakeholder groups have more experience in one approach than another, resulting in a less integrated approach. Hybrid designs are also more complicated to execute [20]. These risks are minimized by the fact that the study team has broad representation of expertise in experimental designs, implementation research, and quantitative and qualitative methods. The clinical team is also committed to providing insight and feedback in all phases of the study and ensuring that study results are useful to clinical decision makers.

The study is also limited by the fact that, while the experimental design is powered to detect differences on a range of important outcomes, it is underpowered to detect statistical difference on some outcomes including fetal or maternal death, low birth weight, or the receipt of standard prenatal testing and care. Furthermore, although reducing office visits and healthcare utilization can yield cost savings, realizing any cost gains will require structural adjustments to staffing models. If practices fail to capitalize on the opportunities for infrastructure change, the OB Nest program could improve patient and provider satisfaction without realizing the potential economic gains. Finally, this study is limited by its focus on low-risk patients, but in-depth reporting on a range of outcomes may inform future work with other patient populations.

### Expected impact

We are currently enrolling patients in this study. Upon completion of this study we will have gained an understanding of whether this new model of prenatal care improves patient-reported outcomes, supporting the clinical team’s theory of action related to what women want in prenatal care. It will also provide evidence on whether this model is safe and effective in changing the paradigm of health care utilization toward interventions delivered by expert registered nurses and away from provider-focused care. This is a critical factor as health care institutions endeavor to improve care while controlling costs.

This study has the potential to increase uptake of new models of reduced visit care, which has faltered even since the Expert Panel on the Content of Prenatal Care recommendation in 1989. Hybrid designs and evaluation frameworks like RE-AIM focus studies like this on external validity and the translatability of evidence-based practice by including methods to understand implementation [20, 25, 38]. We also aim to add to the growing literature on embedding process evaluations and qualitative methods in RCTs [39, 40] by addressing the value of these methods starting in pre-trial planning and fully reporting how each method is to be used and why each was selected.

## Additional file

Additional file 1:
**Title of data: Patient enrollment exclusion criteria.** Description of data: List of high-risk factors that would disqualify patients from participation. (PDF 86 kb)
