# Supplementary material for: Implementation of a new prenatal care model to reduce office visits and increase connectivity and continuity of care: protocol for a mixed-methods study
Source: BMC Pregnancy Childbirth. 2015 Dec 2;15:323. doi: 10.1186/s12884-015-0762-2 (PMC4668747; doi:10.1186/s12884-015-0762-2)
Supplement: Additional file 1: — Title of data: Patient enrollment exclusion criteria. Description of data: List of high-risk factors that would disqualify patients from participation. (PDF 86 kb) [file 12884_2015_762_MOESM1_ESM.pdf]

Additional file 1. Patient enrollment exclusion criteria

Exclusion Criteria:

1. Clinical judgment that determines that the pregnancy is at high risk for complications Any of the following high risk factors would disqualify the mother for the study:
    - i. 37 years of age or greater
    - ii. Severe hypertension (>160/110)
    - iii. Possible ectopic
    - iv. Congenital adrenal hypertension
    - v. Prior PE/DVT/stroke
    - vi. Anticoagulation during prior pregnancy (e.g. antiphospholipid antibody syndrome)
    - vii. Prosthetic heart valve (non-bio)
    - viii. Pulmonary hypertension
    - ix. Mothers currently taking Immunosuppressant's, Prednisone > 10mg per day, antipsychotic (e.g. lithium, Haldol, Zyprexa), chemotherapy
    - x. Recurrent pregnancy loss (>2 losses)
    - xi. Current maternal malignancy
    - xii. Prior myocardial infarction/cardiomyopathy
    - xiii. Bio-prosthetic heart valves
    - xiv. Marfan syndrome
    - xv. Active liver disease (e.g. hepatitis)
    - xvi. Congenital heart disease
    - xvii. Coagulopathies including thrombophilias and bleeding disorders.
    - xviii. Pre-existing diabetes
    - xix. Genetic disease/CF testing/anomalies in prior child
    - xx. Incompetent cervix (prior cerclage)
    - xxi. Twins, Triplets or quads diagnosed by REI or ultrasound.
    - xxii. Isoimmunization (Rh, Kell, etc.)
    - xxiii. History of transplant or currently on Dialysis
    - xxiv. Recurrent pregnancy loss (antiphospholipid antibody syndrome or collagen vascular disease)
    - xxv. Chronic hypertension
    - xxvi. Prior 2nd or 3rd trimester loss
    - xxvii. HIV
    - xxviii. Inflammatory bowel disease
    - xxix. Asthma and currently on steroid to control disease
